# Editorial: Surviving and thriving: how crops perceive and respond to temperature stress

**DOI:** 10.3389/fpls.2024.1550257

**Published:** 2025-01-10

**Authors:** Zemin Wang, Yi Wang, Darren Chern Jan Wong

**Affiliations:** ^1^ State Key Laboratory of Aridland Crop Science, Gansu Agricultural University, Lanzhou, China; ^2^ College of Life Science and Technology, Gansu Agricultural University, Lanzhou, China; ^3^ State Key Laboratory of Efficient Production of Forest Resources, Yinchuan, China; ^4^ Beijing Key Laboratory of Grape Science and Enology, and Chinese Academy of Sciences (CAS) Key Laboratory of Plant Resources, Institute of Botany, Chinese Academy of Sciences, Beijing, China; ^5^ Division of Ecology and Evolution, Research School Research of Biology, The Australian National University, Acton, ACT, Australia; ^6^ School of Agriculture, Food, and Wine, Waite Research Precinct, University of Adelaide, Adelaide, Australia

**Keywords:** abiotic & biotic stress, transcript factor, tolerance, extreme temperature stress, crop

Climate change-driven temperature fluctuations pose a significant threat to crop productivity, emphasizing the crucial need to elucidate how plants mitigate temperature stress-induced cellular damage to ensure agricultural sustainability and food security (Jin et al., 2024; Ding and Yang, 2022; Shi et al., 2018, 2015; Zhu, 2016). In this Research Topic, “*Surviving and Thriving: How Crops Perceive and Respond to Temperature Stress*,” 12 original research articles have presented new and valuable insights into the genetic basis of temperature extremes (e.g. cold/freezing and/or high heat) in a wide range of cereal (e.g. wheat, rice, pearl millet), vegetable (i.e. pepper), legume (e.g. soybean), and fiber (e.g. cotton) crops, paving the way for enhancing crop resilience and contributing to global food security, with potential biotechnological applications ranging from textiles to biofuels. We briefly summarize these contributions below.

## Frost-tolerant wheat varieties reveal key molecular pathways conferring cold tolerance

Spring frost poses a serious threat to wheat production and grain quality, however, the molecular mechanisms conferring frost tolerance remain poorly understood. By taking advantage of a frost-tolerant wheat cultivar and subjecting it to freezing stress at the meiotic stage, Yao et al. revealed that key phenotypic changes (e.g. reduction in plant height, seed setting rate, and cell wall thickening in the stem vascular tissue) coincide with transcriptomic changes impacting hormone signaling, cell wall biosynthesis, and transcription factor regulation pathways.

## Machine learning prioritizes key cold tolerance genes in rice

By leveraging comparative transcriptomic analysis of chilling stress and recovery in resistant and susceptible rice cultivars, Zhang et al. successfully inferred various time-series weighted gene co-expression networks associated with chilling resistance and susceptibility. Several biological processes, including abscisic acid responses, water deprivation responses, protein metabolism, and transcription regulation, were significantly enriched. Using machine learning methods, five genes (e.g. C-repeat binding factor, OsCBF3) were further prioritized to be pivotal for chilling resistance in rice.

## BREVIS RADIX genes: evolution and function in upland cotton’s abiotic stress tolerance

Genome-wide analysis of the plant-specific BREVIS RADIX (BRX) family in upland cotton revealed 12, 6, and 6 BRX candidate genes in *Gossypium hirsutum*, *G. raimondii* and *G. arboreum*, respectively (Wei et al.). Chromosomal localization and collinearity analysis suggest that segmental duplications were the primary driver of BRX gene amplification. Silencing of abiotic-stress responsive GhBRX.1, GhBRX.2, and GhBRXL4.3 further confirmed their pivotal roles in plant tolerance to salt and low-temperature stress, extending their function beyond root and shoot growth (Beuchat et al., 2010).

## TIFY TFs mediate cold stress responses via ROS signaling in pepper

Plant-specific TIFY transcription factors (TFs), characterized by a conserved TIFY domain, are known to regulate growth, development, and stress tolerance (Vanholme et al., 2007). In this Research Topic, Wang et al. identified 16 TIFY plant transcription family candidate genes dispersed across seven subgroups in pepper (Capsicum annuum L.). In particular, *CaTIFY7* and *CaTIFY10b* were highly induced in leaves during cold stress. Knockdown of *CaTIFY7* and *CaTIFY10b* compromised cold tolerance, while their overexpression enhanced it, by influencing many cold-responsive and reactive oxygen species (ROS)-related genes.

## New insights into temperature management strategies for improved soybean production

Ding et al. investigated the impact of five physiologically relevant day/night temperature regimes in two thermotolerant and thermosensitive genotypes. A comprehensive evaluation of physiological, biochemical, and genetic analyses revealed that 35/27°C (T_4_) was the optimal regime for growth and yield in their study system. At this temperature, plants showed enhanced photosynthesis, antioxidant activity, and upregulation of key heat stress-responsive genes.

## Comparative genomics reveals the evolution of heat tolerance mechanisms in pearl millet

Singh et al. performed a multi-tissue comparative transcriptomic analysis and revealed distinct genotype (tolerant vs sensitive)- and tissue (root vs leaf)-specific heat-responsive expression. Enrichment of heat shock proteins (HSPs), ROS scavenging, diverse TF families, and secondary metabolism pathways were observed. Interestingly, foxtail millet shared a high degree of collinearity with pearl millet in specific heat-responsive HSP and TF genes, compared to other cereal grains.

## New insights on the mechanism of meiotic heat sensitivity in the wheat male gametophyte

Fábián et al. used cutting-edge histochemical and transcriptomic methods to compare the responses of heat-tolerant and heat-sensitive wheat cultivars to heat stress during male meiosis. Their study revealed pivotal structural and temporal alterations of wheat male meiocytes and tapetal cells and their transcriptional responses triggered by meiosis-timed heat stress. Notably, the heat-tolerant cultivar maintained cytoskeletal integrity and upregulated genes involved in stress response and meiosis, whereas the heat-sensitive cultivar displayed cytoskeletal disruption, impaired meiotic division, and aberrant tapetum development.

## Growth-regulating factors in potato: multi-faceted roles in development and stress responses

Growth-regulating factors (GRFs) are a plant-specific TF family with diverse roles including regulation of plant growth under stress conditions (Omidbakhshfard et al., 2015). In this Research Topic, 12 candidate GRF genes were identified in potatoes (Cui et al.) A systematic analysis of *StGRF* promoter regions identified numerous cis-acting elements associated with plant growth, development, abiotic stress, and hormone responses. Transcriptomic data supported the tissue-specific expression (e.g. stamens, roots, and young tubers) and differential expression of *StGRFs* under abiotic/biotic stress (e.g. heat and *Phytophthora infestans* infection) and hormone treatment.

## Molecular mechanisms of heat stress tolerance mediated by mitogen-activated protein kinase modules in potato

In plants, mitogen-activated protein kinase (MAPK) modules (MAPKKK-MAPKK-MAPK) are activated by various environmental cues, including heat and cold stress, triggering a cascade of cellular signaling events (Bigeard and Hirt, 2018; Manna et al., 2023) In this Research Topic, Zhu et al.; Zhu et al. screened MAPK and MAPKK gene expression in potato (*Solanum tuberosum*) plants cultivated under mild (30°C) and/or acute (35°C) heat stress conditions, revealing that *StMAPK1* and *StMAPKK5* exhibited highly induced and sustained expression profiles. Overexpression of *StMAPK1* modified photosynthesis and preserves membrane stability in response to heat stress by inducing the expression of heat stress genes (*StHSP90*, *StHSP70*, *StHSP20*, and *StHSFA3*) and regulating multiple redox balance and turgor maintenance pathways. Similarly, *StMAPKK5* overexpression/silencing significantly affected both morphological (plant weight/height) and physiological (photosynthesis, transpiration) traits under heat stress by modulating various stress-responsive and antioxidant enzyme genes and associated biochemical defenses.

## New roles of GATA transcription factors and autophagy-related genes in potato heat stress response

Plant GATA (TFs) regulate diverse developmental processes and organ development (e.g. photomorphogenesis, chlorophyll biosynthesis, stomata formation), however, their role in abiotic stress remains relatively unexplored in plants (Schwechheimer et al., 2022). Zhu et al. screened over 50 potato GATA transcription factor (TF) genes for heat stress response and found that StGATA2 was among the few exhibiting strong and prolonged induction. A deeper understanding of autophagy-related genes (ATG) in potatoes were also gained in this Research Topic (Zhu et al.). A systematic analysis of phylogenetic relationships, chromosome distribution, gene structure, interspecific collinearity, and organ- (e.g. leaf, flower, stem, root, stolon, petiole, and tuber) and stress- (i.e. heat) responsive expression was performed on six putative ATG18 subfamily members identified in the potato genome. *StATG18a* emerged to be highly induced by heat stress in several organs. *StGATA2-* and *StATG18a-*overexpression also elicited comparable phenotypic, biochemical, and genetic responses to heat stress atypical to transgenic potato lines overexpressing *StMAPK1* and *StMAPKK5*.
